# Cases Illustrative of the So-called “Congestive Fever,” or Cerebro-Spinal Affection

**Published:** 1863-06

**Authors:** J. Baxter Upham

**Affiliations:** Surgeon in Charge of Stanley Gen. Hospital, Eighteenth Army Corps


### SELECTED.

CASES ILLUSTRATIVE OF THE SO-CALLED

### "CONGESTIVE FEVER," OR CEREBRO-SPINAL AFFECTION

WHICH HAS RECENTLY APPEARED IN THE CAMPS IN AND AROUND THE TOWN OF NEWBERN, N. C.

By J. BAXTER UPHAM, M. D., Surgeon in Charge of Stanly Gen. Hospital, Eighteenth Army Corps.

I propose, in the present paper, to give, in outline, a few brief notes of the cases of this affection as it has presented itself in hospital during the last two or three months. The disease (so far as I can learn) has, in every case, originated in the camps adjacent to the town, whence it has been brought into the hospital at an earlier or later stage of its progress. The records here submitted are from the note-books of my associates on the medical and surgical staff of the Hospital, and the autopsies were made—under my own inspection mostly—by the attending surgeons in whose wards they occurred.

The four cases first adduced were in the service and under the care of Assistant Surgeon J. Q. A. Meredith, of the 103d Regiment Penn. Vols.

Case I.—J. M., a private, aged 19, was admitted to Hospital on the evening of January 16th, in a moribund condition. No previous history of his case could be obtained, further than that he had been attacked the night before, suddenly and violently. When admitted, he was unconscious, having frequent epileptic spasms; frothing at the mouth; pupils insensible to light. Tonics and stimulants, in large doses, were prescribed, and stimulating injections were administered; these last were not retained, but brought away immediately large quantities of hardened fæces. Sinapisms were applied over various parts of the body and limbs.

17th.—No improvement; skin moist and moderately warm; pulse 160, irregular, soft and very compressible; spasms growing worse; stimulants assiduously employed. Died, without a sigh, at 4 P. M.

*Autopsy*, twenty hours after death. *Head*—Investing membranes of the brain were found much congested—the substances of the brain itself slightly so, with a deposit of pus-colored fluid within the ventricles and at the base of the brain, and upon the lobes of the cerebellum. *Chest*—Lungs greatly congested; more posteriorly than anteriorly. Heart normal in size, with a deposit of lymph in both ventricles. Stomach healthy. Liver normal. Spleen of natural size and highly congested. Kidneys and Peyer's glands healthy.

Case II.—J. C., a private, aged 17, was admitted to Hospital Jan. 10th. No statement of his previous condition received. When admitted, he was in a high state of delirium, with a hot and arid skin; tongue dry and almost impossible to protrude; abdomen tympanitic; pulse frequent and feeble; surface of a livid color. He had involuntary discharges from the bowels, of a blackish hue. His respiration was hurried and difficult; extremities cold. Prescribed pil. hydrarg., gr. i. every hour; quinia, gr. ij. every two hours. Hot applications to back, abdomen and extremities. Whiskey and beef-tea.

Jan. 12th.—No abatement in virulence of symptoms. Continued pil. hydrarg., with an equal amount of pulv. ipecac. every hour. Quinia, grs. iij., every two hours. Continued whiskey, beef-tea and hot applications.

13th.—Growing worse; constant muttering and occasional exclamations. Continued treatment, adding neutral mixture and acid drinks, with sinapisms to chest.

14th.—No improvement. Applied blister to chest and back of neck. Quinia, gr. v., every two hours. Morphia at bed time, to check diarrhœa.

15th.—Moribund. Died at 4 P. M.

*Autopsy*, twenty hours after death. Body small, spare, somewhat below the medium size; external appearance of a dark livid hue. *Head*—Upon removing the calvarium, the investing membranes of the brain were found to be somewhat congested. On cutting into the substance of the brain, some points of blood exuded, more prominent than natural. The arachnoid presented a slightly clouded appearance. In other respects normal. *Chest*—Heart normal; lungs greatly engorged, more so in posterior and dependent portion, where were found circumscribed spots of discoloration, varying in size from that of a split pea to a five-cent piece, resembling somewhat the condition of pulmonary apoplexy. The same appearance, though less marked, anteriorly. *Abdomen*—Spleen nearly double its natural size, greatly engorged with blood. Liver slightly enlarged and somewhat congested. Kidneys normal. Stomach healthy. Peyer's patches in a few instances more prominent than natural, friable, apparently, with, in one case, ulcerative points; not having, however, the legitimate appearance recognized in typhoid fever. Other organs healthy.

Case III.—C. B., private, aged 18, was admitted into Hospital Jan. 16th, in a moribund condition. He had been on duty the day previous, and was attacked in the afternoon with chills, headache and delirium. Cups were applied to the back of the neck, and cathartics and quinine were administered freely. Upon entering the hospital, his breathing was irregular, difficult and accompanied with groaning; 44 inspirations in a minute. His pulse was imperceptible. There was a mottled appearance of the skin, approaching petechia. Percussion of chest clear anteriorly; the extremely low condition of the patient precluded percussion posteriorly. Directed enema of spts. vini gallici, ℥iv.; ol. terebinth., ℥i. M. Whiskey and quinine to be given freely. Sinapisms applied to back of neck, spine, abdomen and extremities.

The patient died at 4 o'clock P. M.

*Autopsy*, twenty hours after death. Body of medium size and well developed. *Head*—Brain but slightly congested in its substance, having at its base a deposit or exudation of tenacious consistence, presenting much the appearance of false membrane, conjoined with a pus-colored fluid, most noticeable and abundant around the origin of the nerves of sense and on the base of the cerebellum. The same substance also occurred in the ventricles. No alteration in the texture of the brain itself. *Chest*—Lungs extensively congested, more posteriorly than anteriorly; the upper portion interspersed with tuberculous deposits; the lungs were generally crepitant, with well-defined spots, resembling those of pulmonary apoplexy. Heart normal in size, with extensive deposits of lymph in both ventricles. Old adhesions of both lungs to pleura, and of left to diaphragm. *Abdomen*—Liver enlarged and congested. Stomach healthy. Spleen enlarged to double its natural size, and greatly congested. Kidneys normal. Intestines healthy. No alteration of Peyer's patches.

Case IV.—S. P., a private, aged 32, was brought to Hospital Jan. 16th, in a moribund state. No history of his previous condition given, though we learned from his comrades that he was attacked suddenly the day before. Quinine and whiskey, with capsicum, administered in free doses. Brandy and spirits of turpentine given in enema.

Jan. 17th.—Patient this morning was lying on his right side, his head thrown back, groaning heavily, giving indications of great pain. He was roused with difficulty. His skin was moist and moderately warm; sordes on teeth; tongue dry and inclined to black; pulse 120, weak and compressible, inclined to intermit; petechial eruption on limbs; percussion good in front, flat posteriorly. Treatment of previous day continued. Sinapisms also applied to back of neck and chest, abdomen and calves of legs.

Evening.—Growing worse; powers rapidly failing; totally unconscious and insensible. Same treatment continued with more vigor, accompanied with frictions along the spine.

Jan. 18th.—Died at 7 A. M.

*Autopsy*, six hours after death (conducted by Dr. Fisher, Assistant Surgeon of the 44th Mass.) Cadaver of good size, well developed; no emaciation. Rigor mortis very strongly marked. A few petechial spots are to be seen upon the arms and hands. *Head*—On removal of the calvarium signs of congestion were apparent, the veins of the investing membranes being considerably engorged, beneath which a purulent lymph-like substance was observed, thinly spread over the surface of the brain—upon the base and between the lobes of the cerebellum particularly—and, in greater abundance, about the origin of the nerves, and upon the surface of the medulla oblongata. The same substance was also seen in the lateral ventricles, being here more opaque and thickened than elsewhere. The substance of the brain itself was apparently normal. *Chest*—Lungs moderately congested, more posteriorly than anteriorly, crepitant throughout; old adhesions upon the left lung. Upon opening the pericardium, an abundance of diffluent lymph, easily washed away with water, was observed; its inner surface uniformly congested. The ventricles of the heart were filled with dark fluid blood, which subsequently clotted in the basin. There were no fibrinous deposits; size and texture of heart normal. *Abdomen*—Stomach not examined. Liver normal in size and appearance. Spleen enlarged and slightly softened; of a deep maroon color. Kidneys normal. Small intestines, in the space of two feet from cœcum, showed Peyer's glands, in one or two instances, more prominent than natural; in one case, some loss of substance, not amounting to ulceration; one or two patches a little raised or thickened perhaps. No redness or congestion of the mucous membrane.

The cases which follow occurred in the wards under the charge of Dr. J. B. Treadwell, Ass't. Surgeon of the 45th Mass. Vols.

Case V.—J. M., a private, aged 21, was received into Hospital on the 14th of January, in an algid condition, exhausted and delirious. The statement of his regimental surgeon, Dr. Robert Ware, is to the effect that he was seized, on the 12th, with a chill, followed by high febrile excitement, with a full pulse, and, at first, a hot skin; soon after became delirious. He had been treated with full doses of quinine, the free administration of stimulants, and cupping at the back of the neck. A similar line of treatment was continued on his admission to the Hospital.

Jan. 15th.—Passed an unquiet night, with but little sleep. There was constant muttering delirium and jactitation; pulse 80, very weak; skin cool and moist; respiration quiet; bowels open; marked subsultus tendinum.

Evening.—Symptoms much the same, but aggravated.

Died at 12 o'clock, midnight.

*Autopsy*, twelve hours after death. Body well developed; no emaciation; rigidity about as usual. *Head*—Both ventricles of the brain were greatly distended with a semi-opaque fluid, having a pus-like deposit at the bottom. This same deposit was also manifest at the base of the cerebellum, and entangled about the origin of the nerves of sense, where it was abundant, lymph-like, somewhat tenacious, much resembling false membrane in appearance, and could be traced onward, by carefully unfolding the convolutions, into both lateral ventricles. *Chest*—About three ounces of fluid were found in the pericardial cavity. Heart below the normal size; mitral valves a little thickened; very firm adhesion of the pleural surface throughout the whole of the left side. Right lung, with the exception of a small portion of anterior lobe, congested. Left lung one-third smaller than natural—the result of disease. *Abdomen*—Liver of natural size; external appearance good; interlocular veins somewhat congested. Spleen normal. Kidneys healthy. Stomach slightly congested, with cadaveric softening along its greater curvature. Peyer's patches appeared slightly engorged and enlarged, and were more prominent than natural.

Case VI.—G. B., a private, aged 21, was received into Hospital in evening of the 19th of January. The statement of his regimental surgeon, Dr. Robert Ware, was as follows: "Patient was attacked suddenly with symptoms of a severe cold, and some disposition to paralysis of the tongue and muscles of the face. He was treated with quinine in half-drachm doses, stimulants and beef-tea, and was cupped to the extent of five ounces at the back of the neck. Reaction came on, though incompletely.

"Jan. 19th.—Same treatment continued, but he has become gradually worse, and, since noon, delirious."

Evening.—Patient, on admission into Hospital, appeared in a state of complete exhaustion, almost amounting to collapse, death following immediately.

*Autopsy*, twelve hours after death. Body of medium size, but little emaciated. Rigor Mortis great. Arms, chest and legs studded with petechial spots, of one, two and three lines in diameter. *Head*—The membranes of the brain but little, if any, congested. Slight cloudiness on the superior surface visible through the membranes; a little opacity of the arachnoid noticed; otherwise nothing abnormal. *Chest*—Lungs more than usually engorged, especially at dependent and posterior portions; crepitant throughout. The pericardium contained six or eight ounces of sero-purulent fluid, holding in suspension masses of flocculent lymph; both surfaces covered with a layer of lymph, of sufficient thickness and consistency to be torn off like a membrane, having the appearance, in fact, of genuine false membrane; some fibrinous clots in the ventricles, and thickening of the mitral valves. *Abdomen*—The liver, spleen and kidneys were normal; mucous membrane of stomach and intestines also natural. Peyer's patches healthy.

Case VII.—O. W. W., a private, aged 22, was suddenly attacked, according to the statement of his regimental surgeon (Dr. Newton), on the 13th of January, with violent headache, accompanied with hot skin and full pulse.

14th.—All symptoms aggravated, with retention of urine.

Jan. 15th.—Admitted to Hospital in a state of high febrile excitement; pulse 70, full and moderately strong; hot skin; dusky hue of face; easy respiration; much delirium, patient frequently attempting to get out of bed. Prescribed wine, ℥i. every two hours; quinine, gr. vi. every three hours; also, hyd. chlor. mit., gr. xv., to be taken at once, to be followed in three hours with carb. ammon., gr. v. Delirium still increased; skin became cool and moist, with less febrile excitement. [The further notes of this case have been mislaid.] All symptoms, however, became rapidly aggravated; delirium gave way to symptoms bordering on coma; the respiration became hurried; involuntary discharges increased, and the patient died, with but slight signs of exhaustion, on the 22d of January.

*Autopsy*, four hours after death. Subject of medium size. No petechiæ (a few spots had appeared, during his illness, upon the left forearm). No rigor mortis. *Head*—Dura mater healthy; upper surface of brain slightly engorged; on the base of the brain, around the origin of the nerves of sense, and upon the medulla oblongata especially (sheathing this latter completely), was observed a deposit of consistent, pus-like lymph of about two lines in thickness, extending also into the crevices of the brain and cerebellum. On opening the ventricles, a gush of dirty, semi-opaque fluid exuded, and, at the bottom and posterior parts, flakes of lymph were seen. The right lateral ventricle contained, in its posterior part, about a drachm of this lymph-like matter. *Chest*—Heart normal. The left lung of slate color, and has on its posterior aspect a hepatized look and feel. Bronchiæ filled with tenacious lymph-like substance, of a consistency sufficient, in some parts, to be drawn out by the forceps. The lung, indeed, was in a state of red hepatization. *Abdomen*—Stomach healthy. Liver of normal size, a little congested. Gall-bladder distended with dark fluid bile. Kidneys natural. Spleen very small, not congested, of lighter color than natural. Peyer's patches natural.

Case VIII.—D. N. H., a private, 18 years of age, was admitted to Hospital on the 13th of January. His previous circumstances and condition, according to the statement presented by his regimental surgeon, Dr. Kneeland, were as follows: "Patient was of nervous temperament and of rather slight figure—had always enjoyed good health up to the present time. In the forenoon of January 28th, had a slight chill, which was succeeded by violent headache and pain in back and limbs; he had slight epistaxis; urine was scanty and high colored. ℞. Ol. ricini, ℥ i. ℞. Spts. eth. nit., ʒ i., every two hours.

"29th.—No better; all symptoms increased in severity; complains of ringing in ears; pulse full and frequent; tongue furred; some cough. Treatment continued."

30th.—Had, on admission to Hospital, the following symptoms: Skin hot and dry; severe headache, particularly in occipital region; head thrown back; tongue dry, and dark colored in centre, with white edges; abdomen natural; extremely violent delirium. ℞. Liq. ammon. acet., spts. eth. nit., aqua camph., aa ℥ i.; chloroform, ʒ ss. M. S. ʒ ii. every two hours. Sinapisms to feet. Beef-tea and farinaceous diet.

31st.—Rather more quiet. Symptoms, with the exception of delirium, quite as marked as yesterday. Rather profuse epistaxis. ℞. Ol. terebinth., ʒ iv.; syr. simp., ℥ i. M. S. ʒ i. every three hours.

Feb. 1st.—No better; slight diarrhœa; pulse 124, less full. ℞. Quiniæ sulph., ʒ ss.; acid. sulph. arom., gtt. xv.; aqua font., ʒ x. M. S. ʒ i. ter in die.

2d.—Delirium less active; expresses himself better; diarrhœa increased. ℞. Plumbi acet., gr. xviii.; opii pulu., gr. v. M. Div. in pil. No. vi. S. One after second discharge.

3d.—About the same as yesterday; cough rather troublesome; sibilant rales heard over both breasts; sordes on teeth. ℞. Pulv. ipecac. es opii, ʒ ss. Div. in ch. No. 6. One every four hours. Continue treatment.

4th.—Febrile symptoms very much diminished; pulse 100, weak; skin cool and moist; answers quite readily. Omit prescription of January 30th. ℞. Vin. alb., ℥ i., every four hours. Continue treatment.

5th.—Much the same as yesterday; gurgling in the right iliac fossa. Continue treatment.

6th.—Seemed rather stupid; muttering delirium; no diarrhœa. Omit prescriptions of Feb. 2d and 3d.

7th.—About the same; rather weaker; several spots on abdomen. Increase wine to ℥ iss. every three hours.

8th.—Comatose; does not answer questions. Continue wine, turpentine and quinine.

9th.—No better; pulse 90, rather weak; tongue discolored, dry and thickly furred. Continue treatment.

10th.—Died at 4 o'clock P. M., apparently but little exhausted.

11th.—*Autopsy*, fourteen hours after death. *Head*—Slight injection of vessels of cerebral membrane. Lateral ventricles of brain distended with fluid mixed with a purulent-looking substance. On the inferior aspect of the cerebellum and medulla oblongata was a copious deposit of lymph, from one-fourth to three-eighths of an inch in thickness, having a lobulated appearance and being quite firm. There was also a slight deposit of lymph in some of the fissures. *Thorax*—Heart normal. Lungs healthy, with exception of slight congestion of posterior lobes. *Abdomen*—Liver, stomach, spleen, pancreas, kidneys and bladder natural in size and healthy. Peyer's glands very much thickened, and, in one or two instances, ulcerated. The solitary glands somewhat enlarged.

Further illustrations will be given in a future paper.

In its *mode of attack*, the disease was commonly sudden and without premonition, the patient, for the most part, continuing on duty and making no complaints till the very day of his seizure. Some of the most violent cases thus commenced; Case XII, previously cited, is in point, where the soldier appeared with his company at the evening dress parade, complained of chilliness, headache, &c., during the night, and was dead within thirty-six hours following. And the subjects of the disease, in most cases, were those previously in the fullness of robust health—between the ages of 18 and 24—who had endured hardships and exposures with impunity.

The *symptoms* were, at the first, headache, referred oftentimes to the back part of the head particularly, with dizziness —pain in the back and limbs, this last occasionally of an excruciating character—with sometimes rigors, and nausea and vomiting. Chilliness, rather a well-defined chill, characterized the accession of the disease. A peculiar stiffness in the muscles of the face and neck was often an early symptom; this would be followed by local spasms, perversion of vision, &c. In some cases the initiatory symptoms were those of a severe cold, with a disposition to paralysis of the tongue and a portion of the muscles of the face. With this the respiration would be difficult and irregular, giving occasion to fear a congestive attack of the lungs. There was often tenderness at the nape of the neck and along the spine early in the disease. The skin was usually moist, but hot. The face was suffused —often of a dusky hue—and the features distorted in the manner before mentioned—the eyes congested and suffused. There was not, for the most part, active delirium—but perversion of intelligence rather, and dullness and indifference to outward objects, from which condition the patient could be roused and made to answer questions consciously. The tongue had, at first, a white creamy coat, which, in the course of the disease, became yellowish or brown at centre and base, more rarely dry and cracked towards the close. There was loss of appetite, but usually not very urgent thirst. The heart's action was irregular, sometimes tumultuous, to which the pulse did not always respond, being mostly accelerated, but not strong—occasionally intermittent. The bowels were regular, or inclined to diarrhœa and costiveness by turns. Petechiæ were not an unfrequent manifestation—in appearance almost identical with the true typhus eruption, and like that seen upon every part of the body except the face—persistent on pressure, varying in hue from the darkest aspect of the measles to that of the true petechial spots imbedded in the skin. Purpural spots, abundant and of large size, were sometimes present, and were always a grave symptom. There was no marked tenderness of the epigastrium or abdomen. In the cases of longer duration, there was, in the last stages, sordes on the teeth and lips, and involuntary evacuations of urine and fæces. The patients often die without much symptoms of exhaustion. The decubitus was mainly on the side, with the head not unfrequently thrown back—the neck rigid and stiff

—a partial opisthotonos. There was uniformly great restlessness and jactitation. As an accompaniment and occasionally a sequel to the disease, iritis was several times observed. So, also, was synovitis—and, in one instance, pericarditis. The above are among the more prominent and constant symptoms —but there was a considerable diversity in the manifestations of the disease during its progress, whether towards a favorable or fatal result; in no one case do I remember to have seen even a majority of those I have enumerated present.

Singular and anomalous symptoms were sometimes noticed. Dr. Jewett, Surgeon of the 51st Mass. Regt., to whom I am indebted for a clear and able account of the disease, as it occurred in the troops under his care, reports that, "in a single case, a pleasing delirium was noticed, with loquacity and decidedly erotic desires, accompanied with priapism more or less extensive during the greater part of the disease." This peculiarity, he adds, was noticed in about one-third of his cases. Dr. Cowgill alludes to the same fact. Dr. Jewett noticed the decubitus upon the dorsum among fourteen cases which occurred in the 51st Mass. Reg. in but a single instance. "In all the others," he observes, "the patients lay upon the side till near the close of life." "In a few cases, and those the most severe ones," he also remarks, "no moan or sound of any kind escaped the patient, but there was a fearful restlessness which ceased only at death; in others there was much moaning." Stiffness of the muscles of the face, before alluded to, amounting at times to spasm, was almost pathognomonic. In some form, this affection was present in nearly all the cases sent in by Dr. Ware; it was common in those treated in Academy Hospital. Dr. Jewett speaks of it as being present in fully one-third of the cases which came under his observation, "there being," as he says, "more or less stiffness of the muscles of the neck and back, with opisthotonos—in one case paralysis of the glosso-pharyngeal nerve, and in two others eversion of the eyes and occasional squinting."

The *duration* of the affection varied from a period of less than thirty-six hours, to that of three, four or six weeks, and even longer. According to my own observation, the more usual duration has been from three or four to seven days.—*Boston Medical Journal.*

